# Supplementary material for: A Likelihood Approach for Real-Time Calibration of Stochastic Compartmental Epidemic Models
Source: PLoS Comput Biol. 2017 Jan 17;13(1):e1005257. doi: 10.1371/journal.pcbi.1005257 (PMC5240920; doi:10.1371/journal.pcbi.1005257)
Supplement: S1 File — (TAR.GZ) [file pcbi.1005257.s014.tar.gz › HSPH_Online-SI-Revision/IPoi09/n1-extreme/n1-extreme_BENCH2_table-4.pdf]

1	newly 3 weeks spec	116.	54.4615	{12., 109.}	53.3422	0.470086	newly 3 weeks cumul	225.	117.923	{30., 249.}	52.841	0.440588	newly 5 weeks spec	137.	80.7846	{24., 114.}	41.0331	0.328584	newly 5 weeks cumul	508.	270.554	{65., 457.}	46.9473	0.379601	newly 64 weeks cumul	896.	840.585	{728., 923.}	7.46909	0.0655393
2	newly 3 weeks spec	95.	92.2	{71., 109.}	12.8722	0.0655745	newly 3 weeks cumul	271.	267.343	{192., 314.}	14.5071	0.076125	newly 5 weeks spec	80.	77.6857	{49., 100.}	19.4643	0.0919927	newly 5 weeks cumul	464.	430.343	{368., 488.}	9.70443	0.0493384	newly 60 weeks cumul	752.	732.	{573., 854.}	11.098	0.0511347
3	newly 3 weeks spec	74.	75.4063	{29., 111.}	33.3193	0.175119	newly 3 weeks cumul	153.	187.859	{74., 286.}	51.5829	0.22615	newly 5 weeks spec	91.	80.4063	{55., 106.}	19.6085	0.106977	newly 5 weeks cumul	361.	349.875	{187., 463.}	24.9134	0.135787	newly 62 weeks cumul	847.	777.922	{638., 892.}	10.5538	0.0508957
4	newly 3 weeks spec	66.	58.2742	{16., 90.}	35.8016	0.230696	newly 3 weeks cumul	162.	143.823	{40., 236.}	41.3481	0.258359	newly 5 weeks spec	58.	67.2581	{43., 87.}	31.2013	0.154515	newly 5 weeks cumul	300.	274.387	{118., 409.}	32.6129	0.19344	newly 61 weeks cumul	732.	768.774	{653., 846.}	9.82725	0.0422943
5	newly 3 weeks spec	89.	46.7541	{30., 64.}	47.4673	0.299786	newly 3 weeks cumul	241.	140.656	{78., 183.}	41.6366	0.258819	newly 5 weeks spec	98.	44.2623	{30., 62.}	54.8344	0.362867	newly 5 weeks cumul	447.	231.738	{152., 297.}	48.1571	0.30103	newly 54 weeks cumul	764.	572.475	{393., 721.}	25.4935	0.140153
6	newly 3 weeks spec	46.	67.1111	{40., 95.}	54.0373	0.182417	newly 3 weeks cumul	105.	221.111	{149., 283.}	112.94	0.317498	newly 5 weeks spec	44.	48.127	{24., 76.}	40.0433	0.176173	newly 5 weeks cumul	246.	328.429	{218., 417.}	40.1407	0.144788	newly 59 weeks cumul	557.	502.54	{290., 706.}	27.1437	0.138191
7	newly 3 weeks spec	75.	95.25	{39., 133.}	46.4167	0.188409	newly 3 weeks cumul	185.	310.578	{188., 420.}	71.2078	0.224952	newly 5 weeks spec	81.	57.3281	{14., 106.}	43.6921	0.304503	newly 5 weeks cumul	355.	443.484	{260., 586.}	36.4569	0.143335	newly 63 weeks cumul	753.	572.219	{275., 812.}	27.2493	0.16923
8	newly 3 weeks spec	76.	55.4576	{30., 78.}	28.3229	0.165995	newly 3 weeks cumul	224.	188.034	{134., 241.}	18.7197	0.102329	newly 5 weeks spec	72.	40.1356	{23., 58.}	44.6328	0.281988	newly 5 weeks cumul	382.	276.915	{187., 360.}	27.518	0.152264	newly 57 weeks cumul	642.	454.864	{258., 646.}	31.5196	0.17966
9	newly 3 weeks spec	86.	87.8269	{32., 130.}	35.9794	0.265265	newly 3 weeks cumul	199.	222.288	{75., 352.}	45.3324	0.307593	newly 5 weeks spec	96.	81.8654	{38., 115.}	25.7011	0.235525	newly 5 weeks cumul	400.	394.327	{215., 525.}	27.8702	0.250604	newly 64 weeks cumul	731.	698.231	{391., 878.}	19.6938	0.225795
10	newly 3 weeks spec	73.	63.1094	{48., 78.}	16.8022	0.0847367	newly 3 weeks cumul	217.	189.484	{145., 227.}	15.5458	0.0802581	newly 5 weeks spec	62.	55.1875	{35., 74.}	21.0685	0.105115	newly 5 weeks cumul	362.	305.938	{256., 354.}	15.625	0.0789035	newly 57 weeks cumul	751.	599.156	{422., 749.}	21.1177	0.111122
11	newly 3 weeks spec	88.	102.582	{75., 137.}	25.8989	0.101878	newly 3 weeks cumul	241.	310.209	{194., 382.}	40.1375	0.157081	newly 5 weeks spec	81.	73.4179	{38., 115.}	30.0719	0.150425	newly 5 weeks cumul	416.	473.03	{380., 568.}	19.5357	0.0786145	newly 63 weeks cumul	680.	687.313	{501., 856.}	15.7946	0.0701075
12	newly 3 weeks spec	65.	47.4032	{6., 97.}	48.0645	0.458813	newly 3 weeks cumul	159.	102.839	{14., 219.}	52.8505	0.541182	newly 5 weeks spec	109.	74.5	{12., 109.}	33.7822	0.388824	newly 5 weeks cumul	387.	240.677	{31., 430.}	43.0858	0.493279	newly 64 weeks cumul	698.	786.242	{710., 913.}	27.3246	0.330785
13	newly 3 weeks spec	49.	54.3571	{36., 69.}	25.2187	0.113779	newly 3 weeks cumul	125.	153.696	{93., 193.}	33.8429	0.136517	newly 5 weeks spec	41.	52.6071	{27., 70.}	39.8955	0.148363	newly 5 weeks cumul	222.	259.804	{173., 323.}	27.3407	0.114213	newly 57 weeks cumul	659.	595.214	{420., 725.}	15.2504	0.0782399
14	newly 3 weeks spec	113.	84.5429	{57., 108.}	25.2845	0.14135	newly 3 weeks cumul	293.	268.7	{208., 321.}	13.5495	0.070636	newly 5 weeks spec	92.	63.0286	{25., 94.}	34.4099	0.214048	newly 5 weeks cumul	501.	410.014	{299., 487.}	18.2692	0.0958237	newly 61 weeks cumul	659.	616.343	{393., 807.}	19.0375	0.0945825
15	newly 3 weeks spec	61.	50.7143	{30., 69.}	24.0632	0.186839	newly 3 weeks cumul	196.	144.304	{81., 204.}	28.3801	0.233969	newly 5 weeks spec	65.	48.9286	{31., 66.}	28.6813	0.205797	newly 5 weeks cumul	338.	243.143	{147., 313.}	28.4341	0.226484	newly 56 weeks cumul	615.	590.375	{408., 746.}	18.435	0.14458
16	newly 3 weeks spec	79.	91.913	{59., 120.}	30.3247	0.130962	newly 3 weeks cumul	198.	279.797	{187., 365.}	52.5399	0.203256	newly 5 weeks spec	51.	71.8986	{37., 106.}	52.7423	0.18335	newly 5 weeks cumul	335.	434.884	{320., 529.}	36.8332	0.143611	newly 63 weeks cumul	677.	672.087	{457., 852.}	16.306	0.0751004
17	newly 3 weeks spec	73.	57.1746	{10., 83.}	28.5062	0.231251	newly 3 weeks cumul	179.	146.794	{18., 232.}	32.9254	0.254154	newly 5 weeks spec	61.	63.4921	{20., 85.}	30.0026	0.1987	newly 5 weeks cumul	321.	272.984	{49., 366.}	24.9468	0.197113	newly 60 weeks cumul	748.	738.365	{628., 843.}	9.74663	0.0673019
18	newly 3 weeks spec	100.	93.338	{62., 120.}	18.6056	0.0923836	newly 3 weeks cumul	211.	272.746	{148., 345.}	41.319	0.160736	newly 5 weeks spec	103.	78.507	{48., 117.}	29.6595	0.166814	newly 5 weeks cumul	426.	437.873	{315., 528.}	14.7094	0.0654001	newly 63 weeks cumul	855.	700.183	{525., 866.}	19.1483	0.100985
19	newly 3 weeks spec	67.	50.381	{1., 116.}	59.8436	0.626768	newly 3 weeks cumul	165.	191.738	{25., 339.}	66.3636	0.444507	newly 5 weeks spec	77.	25.8095	{0., 64.}	69.6351	1.0963	newly 5 weeks cumul	334.	253.619	{27., 511.}	51.2261	0.503349	newly 64 weeks cumul	820.	304.762	{27., 637.}	63.1823	0.752746
20	newly 3 weeks spec	98.	70.9167	{50., 91.}	27.6361	0.154123	newly 3 weeks cumul	274.	217.236	{166., 266.}	20.9905	0.115619	newly 5 weeks spec	84.	56.6528	{34., 81.}	33.879	0.204771	newly 5 weeks cumul	458.	341.875	{264., 419.}	25.3548	0.135318	newly 57 weeks cumul	701.	605.056	{400., 792.}	20.7917	0.106728
21	newly 3 weeks spec	44.	55.5741	{16., 85.}	52.399	0.295259	newly 3 weeks cumul	115.	150.463	{53., 238.}	55.4428	0.310542	newly 5 weeks spec	43.	53.9444	{11., 79.}	52.4978	0.311123	newly 5 weeks cumul	219.	261.296	{92., 366.}	43.1591	0.289922	newly 60 weeks cumul	654.	568.815	{178., 782.}	24.2383	0.261642
22	newly 3 weeks spec	79.	78.7101	{49., 107.}	22.9316	0.109675	newly 3 weeks cumul	241.	267.928	{198., 318.}	19.2555	0.0791122	newly 5 weeks spec	63.	50.8406	{25., 81.}	31.723	0.174965	newly 5 weeks cumul	388.	384.029	{277., 476.}	16.5247	0.0767214	newly 61 weeks cumul	683.	530.174	{331., 716.}	24.8966	0.138246
23	newly 3 weeks spec	72.	93.2899	{57., 126.}	41.0829	0.160581	newly 3 weeks cumul	168.	248.406	{109., 346.}	63.1815	0.227604	newly 5 weeks spec	79.	86.029	{49., 118.}	25.2981	0.110109	newly 5 weeks cumul	346.	425.391	{288., 522.}	31.8338	0.128958	newly 63 weeks cumul	679.	761.145	{597., 889.}	19.0305	0.0778475
24	newly 3 weeks spec	106.	86.0725	{26., 129.}	29.8742	0.19783	newly 3 weeks cumul	236.	193.899	{53., 319.}	36.1889	0.236584	newly 5 weeks spec	137.	101.406	{64., 127.}	25.9812	0.146173	newly 5 weeks cumul	531.	395.116	{171., 534.}	26.7693	0.170763	newly 64 weeks cumul	842.	846.594	{733., 910.}	6.73861	0.0299111
25	newly 3 weeks spec	63.	65.3571	{24., 92.}	28.7755	0.180458	newly 3 weeks cumul	139.	178.614	{54., 266.}	50.4522	0.249428	newly 5 weeks spec	68.	68.7714	{39., 90.}	26.0924	0.161309	newly 5 weeks cumul	276.	317.286	{152., 421.}	32.795	0.191466	newly 61 weeks cumul	696.	715.857	{534., 846.}	14.8851	0.1134
26	newly 3 weeks spec	131.	57.1304	{10., 98.}	56.389	0.494004	newly 3 weeks cumul	280.	132.188	{19., 256.}	53.2039	0.468635	newly 5 weeks spec	153.	76.087	{27., 105.}	50.27	0.380607	newly 5 weeks cumul	589.	274.246	{67., 443.}	53.4386	0.428316	newly 63 weeks cumul	887.	814.696	{672., 909.}	9.15151	0.0752671
27	newly 3 weeks spec	61.	67.3906	{48., 88.}	22.5666	0.0942268	newly 3 weeks cumul	160.	208.156	{157., 260.}	37.2461	0.141261	newly 5 weeks spec	54.	55.4063	{31., 82.}	26.6782	0.118539	newly 5 weeks cumul	277.	327.688	{244., 399.}	25.1354	0.0991314	newly 58 weeks cumul	659.	581.641	{405., 776.}	20.3789	0.102655
28	newly 3 weeks spec	77.	88.3509	{37., 132.}	36.5687	0.154648	newly 3 weeks cumul	250.	279.614	{167., 378.}	27.186	0.112354	newly 5 weeks spec	99.	60.2456	{13., 107.}	42.7255	0.325292	newly 5 weeks cumul	460.	412.719	{233., 538.}	20.1335	0.103867	newly 62 weeks cumul	803.	562.123	{253., 817.}	30.9716	0.189821
29	newly 3 weeks spec	145.	44.8889	{13., 72.}	69.0421	0.633052	newly 3 weeks cumul	304.	117.714	{30., 200.}	61.2782	0.545935	newly 5 weeks spec	113.	54.0635	{29., 78.}	52.1562	0.413853	newly 5 weeks cumul	568.	223.254	{89., 341.}	60.6947	0.522891	newly 59 weeks cumul	875.	713.175	{608., 807.}	18.4943	0.14515
30	newly 3 weeks spec	52.	41.3857	{26., 60.}	30.467	0.164031	newly 3 weeks cumul	145.	147.871	{101., 202.}	22.0394	0.0956433	newly 5 weeks spec	41.	27.6429	{15., 38.}	42.2648	0.24793	newly 5 weeks cumul	245.	209.8	{139., 296.}	25.7259	0.128839	newly 54 weeks cumul	398.	319.157	{201., 468.}	32.3295	0.167927
31	newly 3 weeks spec	115.	65.0577	{14., 118.}	45.7692	0.457646	newly 3 weeks cumul	258.	215.865	{72., 343.}	34.9061	0.295867	newly 5 weeks spec	94.	40.3654	{2., 95.}	60.4951	0.760978	newly 5 weeks cumul	471.	311.577	{75., 513.}	38.6249	0.371604	newly 63 weeks cumul	796.	424.096	{75., 817.}	48.0503	0.48967
32	newly 3 weeks spec	106.	81.5246	{29., 127.}	34.7819	0.225684	newly 3 weeks cumul	229.	222.	{75., 341.}	40.6257	0.215585	newly 5 weeks spec	128.	76.377	{19., 112.}	40.3304	0.339325	newly 5 weeks cumul	487.	376.426	{155., 559.}	28.3738	0.174746	newly 63 weeks cumul	773.	652.639	{248., 871.}	22.0219	0.143122
33	newly 3 weeks spec	60.	45.3898	{14., 72.}	32.2599	0.217004	newly 3 weeks cumul	152.	121.746	{38., 202.}	34.8907	0.227691	newly 5 weeks spec	78.	51.0678	{23., 66.}	35.4846	0.241071	newly 5 weeks cumul	313.	223.017	{91., 339.}	31.7052	0.218307	newly 59 weeks cumul	799.	673.814	{568., 787.}	15.8418	0.0983286
34	newly 3 weeks spec	85.	80.6885	{38., 112.}	27.0203	0.147994	newly 3 weeks cumul	234.	212.836	{73., 318.}	30.0617	0.182257	newly 5 weeks spec	96.	79.2295	{39., 107.}	24.2316	0.142743	newly 5 weeks cumul	427.	373.328	{185., 481.}	19.6414	0.117438	newly 62 weeks cumul	764.	740.574	{517., 894.}	14.37	0.0687693
35	newly 3 weeks spec	73.	98.1719	{61., 131.}	47.3245	0.215651	newly 3 weeks cumul	181.	288.422	{156., 373.}	68.2061	0.269406	newly 5 weeks spec	79.	72.6875	{35., 117.}	33.6234	0.220285	newly 5 weeks cumul	361.	449.688	{324., 544.}	34.3231	0.183355	newly 63 weeks cumul	749.	659.172	{455., 851.}	19.1777	0.158566
36	newly 3 weeks spec	81.	67.1695	{17., 96.}	26.9512	0.197395	newly 3 weeks cumul	195.	182.136	{34., 262.}	31.734	0.213008	newly 5 weeks spec	83.	68.	{40., 91.}	22.3606	0.144012	newly 5 weeks cumul	376.	321.492	{95., 422.}	21.0783	0.150709	newly 60 weeks cumul	755.	720.627	{580., 839.}	11.2381	0.0522298
37	newly 3 weeks spec	78.	54.873	{36., 77.}	30.7896	0.186064	newly 3 weeks cumul	218.	157.937	{109., 204.}	28.2802	0.166596	newly 5 weeks spec	65.	48.5873	{31., 65.}	26.7643	0.154163	newly 5 weeks cumul	380.	258.429	{201., 326.}	31.9925	0.183878	newly 55 weeks cumul	728.	578.016	{424., 735.}	21.8189	0.115352
38	newly 3 weeks spec	93.	82.1831	{57., 102.}	17.6284	0.0888341	newly 3 weeks cumul	234.	248.592	{202., 297.}	15.7217	0.0707229	newly 5 weeks spec	114.	62.5493	{39., 90.}	45.1322	0.28446	newly 5 weeks cumul	462.	386.07	{315., 444.}	16.6972	0.0842849	newly 60 weeks cumul	754.	629.718	{448., 809.}	19.7071	0.102229
39	newly 3 weeks spec	89.	80.3636	{44., 113.}	24.2084	0.16178	newly 3 weeks cumul	179.	209.152	{89., 312.}	41.6624	0.216556	newly 5 weeks spec	117.	78.6515	{58., 102.}	32.7765	0.22032	newly 5 weeks cumul	414.	367.576	{208., 473.}	19.3017	0.149491	newly 63 weeks cumul	855.	745.076	{618., 882.}	14.0333	0.120694
40	newly 3 weeks spec	70.	73.1077	{40., 111.}	34.9011	0.151409	newly 3 weeks cumul	153.	267.8	{187., 356.}	75.6762	0.232403	newly 5 weeks spec	66.	41.2462	{16., 78.}	47.1562	0.314206	newly 5 weeks cumul	299.	366.015	{228., 516.}	34.9678	0.130337	newly 61 weeks cumul	575.	461.4	{264., 722.}	31.0903	0.168775
41	newly 3 weeks spec	45.	49.6667	{26., 71.}	30.2778	0.141237	newly 3 weeks cumul	135.	159.854	{75., 219.}	36.1574	0.162498	newly 5 weeks spec	55.	45.5208	{25., 64.}	26.25	0.145457	newly 5 weeks cumul	248.	253.313	{153., 337.}	22.5386	0.110168	newly 56 weeks cumul	562.	506.292	{333., 715.}	24.3476	0.123815
42	newly 3 weeks spec	88.	75.3182	{21., 132.}	47.314	0.327813	newly 3 weeks cumul	235.	228.5	{78., 356.}	37.3888	0.252938	newly 5 weeks spec	105.	57.9773	{0., 116.}	50.4545	0.672501	newly 5 weeks cumul	462.	354.023	{155., 556.}	34.942	0.278806	newly 64 weeks cumul	862.	526.023	{208., 836.}	39.2771	0.354692
43	newly 3 weeks spec	82.	68.7049	{18., 109.}	33.2067	0.231389	newly 3 weeks cumul	219.	153.541	{46., 247.}	37.2857	0.280803	newly 5 weeks spec	75.	88.0328	{53., 114.}	31.6284	0.134189	newly 5 weeks cumul	390.	321.164	{148., 458.}	26.5868	0.171426	newly 63 weeks cumul	860.	826.721	{747., 906.}	6.65269	0.0305093
44	newly 3 weeks spec	86.	86.9595	{45., 112.}	23.6172	0.130509	newly 3 weeks cumul	240.	231.581	{94., 318.}	28.2827	0.16608	newly 5 weeks spec	76.	80.4865	{55., 105.}	22.0839	0.0993193	newly 5 weeks cumul	408.	398.608	{244., 484.}	18.2068	0.102743	newly 61 weeks cumul	762.	773.446	{645., 877.}	9.54281	0.0416744
45	newly 3 weeks spec	79.	72.6508	{18., 129.}	47.2172	0.314269	newly 3 weeks cumul	203.	164.302	{31., 329.}	48.6199	0.372483	newly 5 weeks spec	105.	91.254	{53., 122.}	22.7967	0.178935	newly 5 weeks cumul	422.	341.079	{114., 529.}	32.9722	0.265417	newly 64 weeks cumul	886.	826.032	{735., 907.}	8.20166	0.0935706
46	newly 3 weeks spec	93.	78.3582	{59., 98.}	18.9857	0.102364	newly 3 weeks cumul	223.	227.97	{139., 282.}	18.8006	0.093319	newly 5 weeks spec	79.	68.7761	{43., 97.}	22.7659	0.122536	newly 5 weeks cumul	409.	371.09	{298., 442.}	13.6554	0.071279	newly 61 weeks cumul	815.	666.015	{481., 821.}	19.4561	0.103364
47	newly 3 weeks spec	63.	65.9385	{25., 99.}	39.1453	0.208842	newly 3 weeks cumul	164.	160.923	{54., 262.}	41.6135	0.231903	newly 5 weeks spec	67.	80.6462	{50., 102.}	31.1596	0.126505	newly 5 weeks cumul	308.	319.169	{130., 444.}	32.048	0.160759	newly 62 weeks cumul	785.	797.323	{681., 890.}	8.74669	0.038205
48	newly 3 weeks spec	60.	57.8627	{9., 106.}	54.7386	0.363461	newly 3 weeks cumul	141.	194.333	{51., 323.}	67.5289	0.311053	newly 5 weeks spec	68.	37.7451	{0., 76.}	54.4694	0.697169	newly 5 weeks cumul	280.	278.216	{64., 464.}	47.1218	0.306318	newly 62 weeks cumul	672.	390.098	{64., 733.}	46.9859	0.435735
49	newly 3 weeks spec	96.	24.	{0., 52.}	76.7857	1.14744	newly 3 weeks cumul	261.	134.524	{30., 294.}	55.1907	0.466885	newly 5 weeks spec	115.	5.59524	{0., 16.}	95.1346	2.09003	newly 5 weeks cumul	493.	151.31	{30., 323.}	69.3084	0.678575	newly 64 weeks cumul	853.	158.69	{30., 327.}	81.3962	0.90196
50	newly 3 weeks spec	57.	5.02326	{0., 8.}	92.1665	1.99769	newly 3 weeks cumul	158.	27.5814	{0., 41.}	88.6959	1.37933	newly 5 weeks spec	54.	1.16279	{0., 3.}	97.8467	2.40714	newly 5 weeks cumul	272.	31.0698	{0., 50.}	89.9795	1.56972	newly 62 weeks cumul	579.	31.5814	{0., 50.}	94.5455	1.89151
